# 

**Published:** 1918-05-04

**Authors:** 


### CONTENTS.

| | PAGE |
|---|---|
| **The Problem of Advanced Consumption** ... | 87 |
| **The Making of a Trained Nurse: Possible Changes Due to the War** ... ... ... | 88 |
| **Hospital and Institutional News** ... ... ... | 89 |
| New Chief Commissioner of Canadian Red Cross—Fulham Palace as a Hospital—The South African Hospital—Pooling Appeals at Sheffield—The Weakness of Vigilance Societies—The Hospital Look—The "Impertinence" of the London County Council—Turn of Fortune's Wheel—The Mint and the Red Cross—Trinkets and Appeals—Educational Lectures to Soldiers—Staff Vacancies at Nottingham—Extension to Queen Mary's Hospital—A Medical Superintendent's Quarters—A Potato Patch for a Hospital—The Disabled as Dental Mechanics—A Subject for Research—Day Sanatoria for London Children—The Mildmay Mission Hospital—Is the Vote Worth the Disclosure?—News from British Prisoners—First Woman Candidate for the Pharmaceutical Council—To Our Readers. | |
| **The Great War** ... ... ... ... ... ... | 93 |
| VI. The Progress of the Sick. VII. Sanitation. | |
| **Botulism** ... ... ... ... ... ... ... | 95 |
| Its Nature, Symptoms and Treatment. | |
| **Rations for Hospitals** ... ... ... ... | 96 |
| **Promotion in the R.A.M.C.** .. ... ... ... | 96 |
| **Human Temperaments** ... ... ... ... ... | 97 |
| The Worthy Person. | |
| **The Institutional Library** ... ... ... ... | 99 |
| A Large Social Experiment. | |
| **The Editor's Letter-Box** ... ... ... ... | 101 |
| The Employment of the Waiting Years—The Site of St. Mark's Hospital—The Shortage of Domestic Help: Work of National Importance. | |
| **Better Conditions of Service Imperative** ... | 102 |
| The Economics of Nursing. | |
| **H.M. Queen Mary, British Mothers, and their Warrior Sons** ... ... ... ... ... | 103 |
| **Round the Hospitals** ... ... ... ... ... | 104 |
| Numerous College Meetings—The Strength of the College Proved—Smoking: Colonial Matrons' Views—Mess Allowances for Army Nurses—A Reformed Fever Hospital—Fine Entry of Probationers at St. George's. | |
| **The Australian Nursing Service in the War** ... | 106 |
| **The College of Nursing** ... ... ... ... | 107 |
| Meeting in Leeds: Local Centre to be Formed. Meeting in Manchester. | |
| **Matrons' and Sisters' Appointments** ... ... | iv |
| **Recent Official Publications** ... ... ... | iv |
| **Parliament and the Profession** ... ... ... | iv |

